# Age-related macular degeneration: genome-wide association studies to translation

**DOI:** 10.1038/gim.2015.70

**Published:** 2015-05-28

**Authors:** James R. M. Black, Simon J. Clark

**Affiliations:** 1 Faculty of Medicine, Sir Alexander Fleming Building, Imperial College London, London, UK; 2 Centre for Ophthalmology and Vision Sciences, Institute of Human Development, University of Manchester, Manchester, UK

**Keywords:** age-related macular degeneration, complement cascade, genome-wide association studies, novel therapeutics

## Abstract

In recent years, genome-wide association studies (GWAS), which are able to analyze the contribution to disease of genetic variations that are common within a population, have attracted considerable investment. Despite identifying genetic variants for many conditions, they have been criticized for yielding data with minimal clinical utility. However, in this regard, age-related macular degeneration (AMD), the most common form of blindness in the Western world, is a striking exception. Through GWAS, common genetic variants at a number of loci have been discovered. Two loci in particular, including genes of the complement cascade on chromosome 1 and the *ARMS2*/*HTRA1* genes on chromosome 10, have been shown to convey significantly increased susceptibility to developing AMD. Today, although it is possible to screen individuals for a genetic predisposition to the disease, effective interventional strategies for those at risk of developing AMD are scarce. Ongoing research in this area is nonetheless promising. After providing brief overviews of AMD and common disease genetics, we outline the main recent advances in the understanding of AMD, particularly those made through GWAS. Finally, the true merit of these findings and their current and potential translational value is examined.

*Genet Med* **18** 4, 283–289.

Unraveling the genetic basis of Mendelian disorders has been a success story of human genetic endeavor over the past three decades. Recent technological progress, in addition to the completion of the International HapMap Project in 2005 (ref. 1), has enabled greater elucidation of the genetic components of common polygenic diseases. The genome-wide association study (GWAS) has been the most common modality used in such investigations. GWAS attempt to identify single-nucleotide polymorphisms (SNPs) that occur more frequently within the genomes of sufferers of a disease than in a control population. It is generally accepted that these variations at single bases within the genome are proxies for a contributory variant, and their locations can therefore be used to infer genomic regions for disease association. In addition, because these associations are typically free from the confounding influences, such as social or behavioral factors, that can plague epidemiological research, they can be used in accordance with the principle of Mendelian randomization as surrogates for exposure when examining the effects of putative causal associations for disease.^2^ GWAS are most effective for common diseases whose causative alleles are derived from a common ancestor within the population and therefore follow the “common disease, common variant” hypothesis.^3^ This view—that disease-causing alleles are common within a population—was especially popular before the first GWAS.^4,5^

Since the first GWAS in 2002, analyzing genetic susceptibility to myocardial infarction,^6^ more than 1,000 others of various sizes have been performed, testing a plethora of common diseases with differing degrees of success. Arguably, the best-known and most successful GWAS was by Klein et al.^7^ in 2005, a landmark study of the most common form of blindness in the Western world, age-related macular degeneration (AMD). This triggered numerous more detailed studies of AMD.

Since that time, GWAS have discovered a vast array of significant variants that have advanced our understanding of the biology of common disease. A striking example is Crohn's disease: Duerr et al.^8^ and Rioux et al.^9^ implicated the interleukin-23 cytokine and autophagy pathways, respectively, in its pathology. Also, Sladek et al.^10^ showed a new role for β-cell zinc transport in type 2 diabetes, and multiple studies revealed new loci causing autoimmune disease.^11^ Nevertheless, GWAS have most frequently uncovered only variants with a small effect—that is, those of low penetrance with small odds ratios (a measure of the odds of a given allele being present in one population sample compared with those of its presence in another sample). In general, as a result, these studies tend to have little value in terms of predicting an individual's risk. Furthermore, for many common conditions with familial components, such as schizophrenia, the majority of their heritability remains “hidden,” most likely in rare variants invisible to GWAS.^12^ Notwithstanding the argument that this may be attributed to inadequate sample sizes, the largest studies, using as many as 250,000 samples, are carried out at great cost and often only minimally increase our ability to explain heritability.^13^

In this review we overview AMD and summarize what is known about its genetic component. We discuss the seminal GWAS and how it has improved our knowledge of the pathogenesis of AMD. Finally, the current and potential future clinical benefits derived from this, such as those granted by disease screening, are analyzed.

## AMD

**Figure 1:**
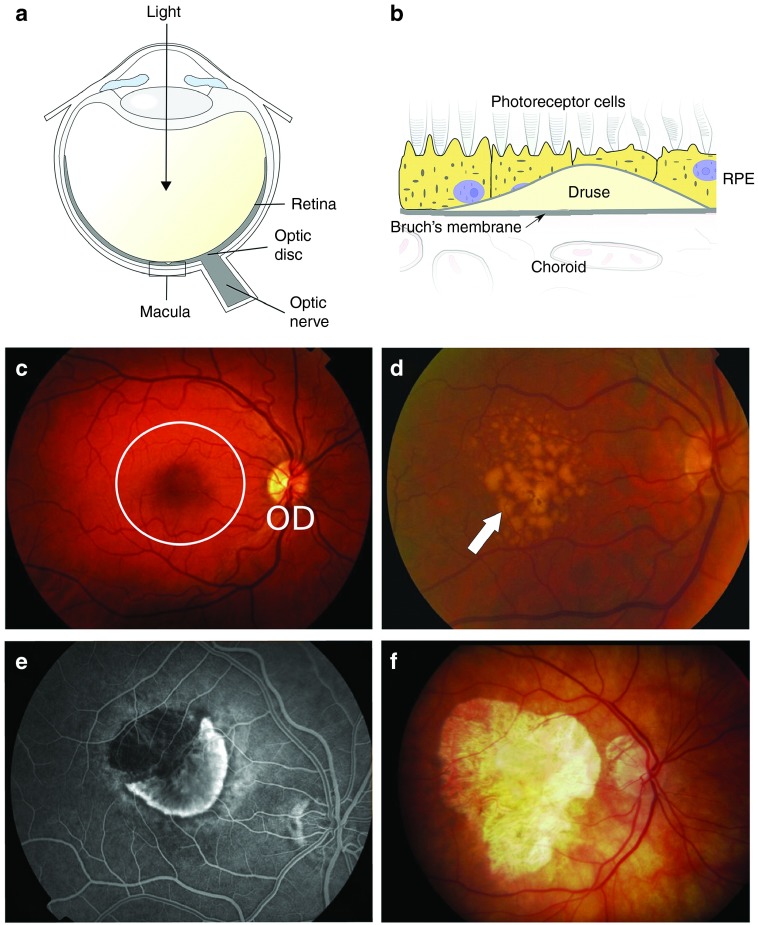
**Changes in ocular phenotype with age-related macular degeneration (AMD) progression.** Retinal images acquired by fundoscopy show the varying stages of disease progression. (**a**) A schematic showing the location of the macular region of the eye where drusen formation leads to AMD. (**b**) A cross-sectional schematic demonstrating that drusen form within Bruch's membrane, which itself is sandwiched between the retinal pigment epithelium (RPE) and the blood supply of the eye (choroid). (**c**) A healthy human eye, in which the radial blood vessels can be seen emanating from the optic disc (OD). The macula region is circled and is 5 mm in diameter. (**d**) In an eye presenting with early stages of AMD, drusen (white arrow) can be seen accumulating in the macula. (**e**) The presence of drusen may lead to choroidal neovascularization resulting in neovascular, or “wet,” AMD. (**f**) Geographic atrophy, or “dry” AMD, where there is complete loss of the RPE layer (seen here as the light yellow region in the macula).

AMD is a progressive disease affecting the central portion of the retina: the macula. Early stages of the disease are characterized by an often asymptomatic accumulation of focal extracellular deposits, representing the classical AMD lesions, termed “drusen,” that form within Bruch's membrane beneath the retinal pigment epithelium^14^ (**Figure 1b**). These drusen contain a barrage of different proteins, inflammatory mediators, and lipids,^15^ although, given the sheer number of candidates found in drusen it is difficult to identify what may act as a nucleating point of formation as opposed to being simply subsumed into the lesion over time. The severe, late-stage form of AMD affects 2.4% of individuals over the age of 50 in the United Kingdom,^16^ and with the population of the developed world aging, this prevalence is expected to increase. Late-stage AMD is subdivided into two types, so-called dry and wet forms (**Figure 1d**–**f**). Dry AMD is more common; well-demarcated “geographic” atrophy of the retinal pigment epithelium and underlying choroidal vessels causes progressive central visual loss. Wet, or neovascular, AMD is associated with severe visual loss and results from choroidal neovascularization breaking through Bruch's membrane (which overlies the choroid) into the neural retina.^14^ These new, fenestrated vessels leak blood, resulting in fibrous macular scarring and causing more acute central vision loss. Although little was known about the pathogenesis of AMD a decade ago, today, thanks in part to GWAS, we have a clearer understanding of its etiology and contributory genetic and environmental risk factors.^17^

## Contribution of GWAS to our understanding AMD

By 2005, AMD was known to be a multifactorial disease with a strong genetic component, but standard analytical methods for single-gene disorders, such as candidate gene analysis and linkage analysis, provided only limited insight. Indeed, although studies examining genes mutated in rare Mendelian macular conditions (e.g., *TIMP3* (Sorsby disease), found links to AMD,^18^ they still represented only a small fraction of the total heritability of AMD.

Early studies examined alleles shared between siblings, attempting to identify broad genomic regions suitable for further research; a meta-analysis of six such studies found evidence of susceptibility loci, notably at 1q and 10q26 (ref. 19). Many of the genetic alterations found on chromosome 1 reside in genes encoding components of the complement cascade, part of a host's innate immune system. Complement dysregulation caused by a complement factor H (FH) mutation, which was known to lead to membranoproliferative glomerulonephritis (often referred to as dense deposit disease),^20^ had also been implicated in AMD pathology, as affected individuals present with retinal drusen.^21^ In addition, a number of histochemical studies sought to analyze the changes that occurred within drusen of affected retinas^22^; crucially, a role for complement activation was highlighted.

In 2005 four separate studies examined SNP associations and found that variation within the *CFH* gene on chromosome 1 represented the most significant predisposition for AMD.^7,23,24,25^ A point mutation, rs1061170, was identified as the susceptibility allele. This rs1061170 SNP results in a coding change in the FH protein, where a tyrosine residue is replaced by a histidine residue at position 402 (or position 384 in the mature protein numbering^26^), referred to as the Y402H polymorphism. Interestingly, ~30% of individuals of European descent carry at least one copy of the Y402H risk allele.^27^ Two studies tested SNPs within target regions of chromosome 1q, previously indicated by linkage analysis studies, for AMD association using case–control populations.^23,25^ In another, Hageman et al.^24^ examined SNPs within the candidate *CFH* gene; they had previously implicated complement activation in AMD pathogenesis.^22^ The most noteworthy of the studies, the GWAS by Klein et al.^7^, examined more than 100,000 SNPs across the whole genome within 96 cases and 50 controls. Even with such a relatively small sample size, it successfully identified a small number of SNPs that were highly significantly skewed within patients with AMD relative to controls; two of these were intronic variants within *CFH*, which proved functional as proxies for Y402H. These initial findings have been irrefutably confirmed by many subsequent successful GWAS.^28^

## Genetic contribution to AMD pathogenesis

FH is a key regulator of the alternative complement pathway, deactivating C3b that has been deposited both on host cells and, crucially, the extracellular matrix, such as the acellular Bruch's membrane. Deposited C3b otherwise activates a host immune response. FH is primarily synthesized in the liver but also is expressed locally in retinal pigment epithelium cells.^24^ The FH protein comprises 20 complement control protein (CCP) domains; the Y402H polymorphism is located in CCP domain 7. The exact causal effects of the many different *CFH* alleles are not yet fully understood. Indeed, since the original study of Y402H by Klein,^7^ other markers in weak linkage disequilibrium with Y402H have shown stronger associations with AMD, although these generally represent variants comparatively less common within the population.^27^ However, given that the CCP 7 region binds C-reactive protein and heparan sulfate (as methods of self-recognition),^29^ and that it is known that the Y402H polymorphism reduces FH binding to both C-reactive protein^30^ and heparan sulfate in Bruch's membrane,^31^ it is likely that this variant leads to a dysregulation of complement. Indeed, deposition of an increasing amount of the terminal membrane attack complex (which is indicative of increased complement turnover) under Bruch's membrane occurred in 402H variant homozygous donor eyes compared with 402Y “risk” donor eyes.^32^ Although unlikely to represent the initiating event of AMD, there is little doubt that a proinflammatory environment, driven by poor complement regulation conferred by the Y402H polymorphism, aggravates drusen formation and contributes to disease progression.^30,33^ A second major locus for susceptibility to AMD, at chromosome 10q26, was identified by a similar combination of targeted linkage studies and confirmatory GWAS.^34,35,36,37^ The mechanisms by which these effects are exerted are less well studied and are confounded by the presence of linkage disequilibrium between two genes, *ARMS2* (LOC387715)^34,35^ and HtrA serine peptidase 1 (*HTRA1*),^36,37^ that lie within a 200-kb region at the candidate locus. To date, the relative importance of each of these genes in AMD predisposition remains contentious. Nevertheless, it is tempting to note that, although the *ARMS2* gene has not yet produced a detectable gene product in vivo, the HtrA1 protein from *HTRA1* is involved in extracellular matrix turnover, especially given the site of drusen formation—Bruch's membrane—forms part of the extracellular matrix.

FH and *ARMS2*/*HTRA1* alleles represent the most influential of all the genetic factors contributing to AMD, and together they increase AMD predisposition by more than 40 times.^38^ Other genes, however—most of which also are involved in the complement pathway—have been implicated through GWAS and candidate studies of genes functionally related to complement.^39,40,41^ The genes encoding complement factor B and component 2 were tested using SNP association case–control studies, which found these genes conveyed significant susceptibility^39^; the same was seen for component 3.^40^ Later GWAS, using larger sample sizes, implicated other genes and pathways in AMD pathogenesis, in addition to confirming the contributions of suspected susceptibility genes. These include another complement gene, complement factor I^41^; genes associated with cholesterol and lipoprotein metabolism, *APOE*, *LIPC*, and *CETP*^42^; extracellular matrix maintenance gene *TIMP3*; the atherosclerotic signaling *FRK/COL10A1* variant^43^; the angiogenesis gene *VEGFA*^43^; and the *TNFRSF10A/LOC389641* region.^44^ The AMD Gene Consortium recently found seven new disease loci: *COL8A1-FILIP1L, IER3-DDR1, SLC16A8, TGFBR1, RAD51B, ADAMTS9*, and *B3GALTL*.^28^ A summary of the most significant known genetic contributions to AMD and their proposed roles in its pathogenesis is included in **Table 1**.

In all, the 19 hitherto described loci conveying susceptibility to AMD are estimated to account for as much as 65% of its heritability.^28^ Even accounting for the significant proportion that remains unclear despite intense GWAS interrogation, this represents massive progress toward unraveling the genetic basis of the disorder, and it differs greatly from the extent to which we understand that of the majority of common diseases.^45,46^ In this respect, GWAS for AMD can be, and are, heralded as highly successful.

## Have GWAS for AMD brought about translational benefit?

It was forecast that GWAS success would convey considerable patient benefit; Wray et al.^47^ claimed that the “value of predictive SNPs could be reaped long before the causal mechanism of each contributing variant can be determined.” To assess this, we discuss the ways in which GWAS discoveries have altered our ability to predict AMD progression.^47^ We then analyze whether we are able to tailor individual therapies to each patient as a result,^48^ as well as describe the progress made toward developing novel therapeutics for AMD.

One aspect of GWAS that many expected to yield significant translational benefit was the use of putative genetic factors to predict and screen for disease (especially in individuals with a family history of the condition),^47^ with a view toward influencing choice of treatment or patient lifestyle. Indeed, a number of models have been designed for predicting the risk of an individual developing AMD^49^; a recent one in particular claimed to be as much as 90% accurate.^50^ This represents remarkable progress and underlines the extent to which GWAS have advanced our knowledge of and ability to test for genetic factors for the condition. The advertised success of these models, especially relative to those for other common diseases, has coincided with the popular rise of commercial ventures such as 23andMe (https://www.23andme.com) and GenePlanet (http://www.geneplanet.com), which are able to assay genetic variants possessed by an individual. In theory, the ability to predict how, and roughly when, an individual will develop AMD would allow the clinician to personalize treatments based on genetic and environmental risk, thus providing the best tailored treatment. A major pitfall, however, is the current lack of interventions available to combat predicted onset of disease. This is exemplified by the National Health Service's UK Genetic Testing Network not offering a test for AMD susceptibility (http://ukgtn.nhs.uk/find-a-test/). Furthermore, the recent warning given to 23andMe by the US Food and Drug Administration^51^ highlights the potential problems of such biomarker screening when no successful intervention exists to modify disease progression. The clinical impact of screening in the case of AMD hinges on the development of viable therapeutics to influence disease progression or the ability of clinicians to recommend effective lifestyle changes to modulate risk.

The substantial contribution of environmental factors to AMD raises the possibility of altering patient lifestyle in response to genetic testing. Smoking, for example, is a well-established risk factor for the condition^52^ and, theoretically, a knowledge of genetic risk might encourage an individual to cease doing so. However, whether an increased risk of developing a chronic disease, when smoking is known to predispose to many other such diseases, would actually influence the lifestyle of a given individual is debatable. For example, Hollands et al.^53^ showed that patients with a familial risk for Crohn's disease, additionally predisposed by smoking, were no more likely to stop the habit than those without. Also, the dietary intake of a number of substances, notably those with antioxidant properties such as the carotenoids β-carotene, lutein, and zeaxanthin and vitamins C and E, is known to affect progression to advanced AMD.^54^ However, studies attempting to prove that modifying dietary intake of such substances is significantly preventive of AMD have so far been inconclusive.^54,55^

The greatest advancement in the clinical approach to AMD has been the introduction of antiangiogenic therapies for wet AMD^56,57^; the most widely used is the vascular endothelial growth factor–inhibiting monoclonal antibodies bevacizumab and ranibizumab.^58^ Wet AMD, the less common form of the disease, affects vision more severely, and, although great benefit has been derived from this therapy, it only halts disease progression and does not prevent onset, nor does it reverse damage already caused to the vision. Furthermore, it is important to note that the implementation of antiangiogenic agents as a method of treating AMD was brought about independent of GWAS. In fact, despite the different pathways recently implicated in AMD pathogenesis, novel interventions that successfully exploit this knowledge remain elusive, and dry AMD remains untreatable.

However, GWAS have identified the importance of complement activation via its alternative pathway (the pathway controlled by FH) in AMD pathogenesis. As such, a number of complement-based therapeutics are currently undergoing clinical trials (see **Table 2**) or are currently in preclinical development. Eculizumab, an antibody against the complement protein C5, was the first logical choice because it was already in clinical use for other complement-mediated disease (e.g., atypical hemolytic uremic syndrome). The use of eculizumab for treating dry AMD, however, failed to affect the progression of geographic atrophy.^59^ This is perhaps unsurprising because this drug targeted the complement pathway at a point downstream of the alternative activation pathway: All GWAS-identified SNPs are in genes whose proteins are involved in an alternative pathway, not the lectin, classical, or terminal pathways.^60^ Similarly, other therapeutics that also target C5—as either an antibody (LFG316; Novartis) or an aptamer-based C5 inhibitor (Zimura; Ophthotech)—are currently in ongoing clinical trials.

A slightly different approach is represented by the drug lampalizumab (Genentec/Roche), an antibody Fab fragment raised against complement factor D. This has great promise, targeting only the alternative activation pathways of complement (the one associated with AMD) and leaving the remaining pathways unaffected, thus providing patients with continued protection against bacterial infections. Phase II trials have been completed, delivering lampalizumab by intravitreal injection for geographic atrophy. While the results remain unpublished, Roche has indicated that efficacy has been seen and a phase III trial is commencing.

Other putative therapies also are under consideration. These include antibodies against properdin and complement factor B, both of which are essential for the activation of complement via the alternative pathway, and even the introduction of specifically designed “mini” complement regulators in an attempt to readdress the imbalance of complement activation in the back of the eye. Given the early stages of such research, assessing how it will translate into the clinic remains difficult, but nonetheless it demonstrates the considerable effort being put into complement-mediated therapeutics for AMD.

## Powerful genetic research to uncover missing heritability

As previously mentioned, ~35% of the heritability of AMD remains undiscovered. Indeed, the inability of GWAS to elucidate the entire genetic component of common diseases is a recurring theme; for some conditions, the vast majority of their heritability remains unknown.^61^ This failure of GWAS to consistently find individually significant genetic risk factors has led to the rise in popularity of a view of common disease genetics opposing the “common disease, common variant” theory. This new viewpoint is known as the “common disease, rare variant” (CDRV) hypothesis, originally put forward before the first GWAS.^62^ CDRV states that genetic factors causing common disease can be rare within a population, and were they to contribute more to the heritability of such conditions, it would correspond to the selective pressures upon such variants within the gene pool.^3^ In other words, there exist variants of great significance that are too rare for GWAS to uncover. The degree to which these two hypotheses prevail seems to differ from one common condition to the next.

It is thought that elucidation of the hitherto unknown genetic component of AMD, possibly caused by rare variants overlooked by GWAS, would help to elucidate the underlying pathogenesis, fueling drug discovery. These variants could be in the form of new loci, exposing novel pathways as important to pathogenesis, or new variants within known loci, helping to elucidate exactly the functional consequences of mutations. Here, the next-generation sequencing era introduces exciting new possibilities; singling out genetic variants in individuals, no matter how rare within a population, will provide a greater range of genetic factors from which to study gene function and disease mechanisms. The potential impact of this type of study for AMD was illustrated by Raychaudhuri et al.^63^ In that study, high-throughput analysis identified a high-penetrance haplotype for AMD, a rare *CFH* variant, and its functional consequences were examined. The potential advantages conveyed to AMD genetics, and indeed common disease genetics, by the accurate and rapid sequencing of human genomes are therefore significant.

Furthermore, targeted therapeutics require full comprehension of the biology of a condition: The new wave of potential therapies for AMD, such as novel attempts to disrupt the angiogenesis pathway, complement inhibitors, and integrin inhibitors,^64^ has resulted from the success of studies involving functional analysis of molecules and pathways. An example is the study of the functional consequences of the common Y402H FH polymorphism and the fact that it alters the ability of FH to regulate complement at the site of disease pathogenesis.^31,65^ Functional studies of this ilk are anticipated to help discover future potential treatments for AMD, and greater knowledge of the “hidden” heritability of AMD will help their success.

## Conclusions

The GWAS for AMD are a much-celebrated scientific advancement. Although these have provided significant insight into the genetic component of the condition, it has been shown here that the translational benefit derived to date, beyond predictive disease susceptibility, has been limited. It goes without saying, though, that in an ever-changing field with much ongoing research, it is fair to expect greater elucidation of the hidden heritability of the condition (especially by next-generation sequencing) and subsequently more effective functional analysis of the mechanisms underlying AMD pathogenesis in the near future. It is hoped that these advancements will facilitate the discovery of novel effective treatments that will revolutionize clinical management of AMD and simultaneously improve the value of predictive genetic screening.

## Disclosure

The authors declare no conflict of interest.

## Figures and Tables

**Table 1:**
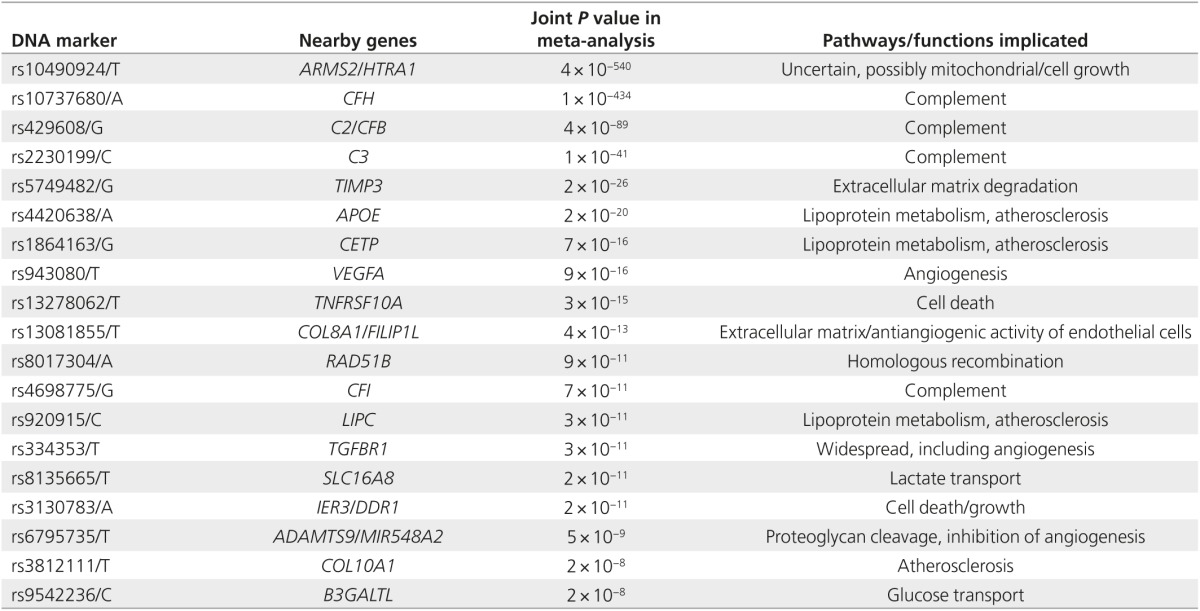
Single-nucleotide polymorphisms associated with age-related macular degeneration and their affected genes

**Table 2:**
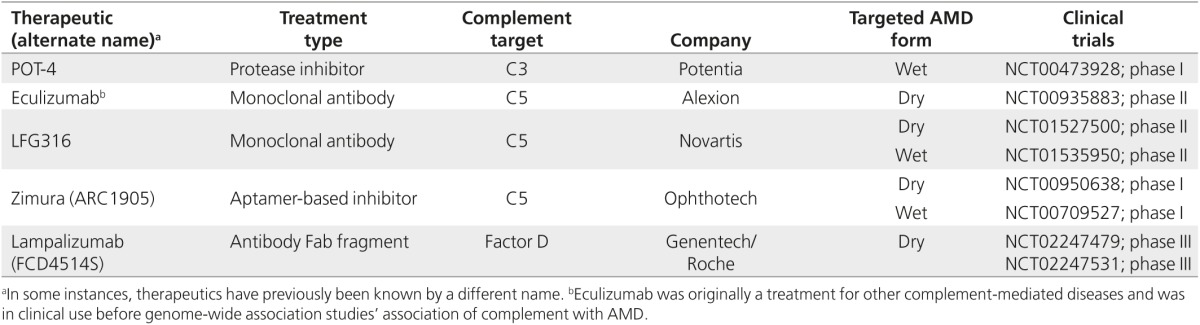
Current complement-based therapeutics directed against age-related macular degeneration (AMD)
